# Cytoplasmic hnRNPK interacts with GSK3β and is essential for the osteoclast differentiation

**DOI:** 10.1038/srep17732

**Published:** 2015-12-07

**Authors:** Xiaoqin Fan, Haiting Xiong, Jinmei Wei, Xuejuan Gao, Yuan Feng, Xiaohui Liu, Gong Zhang, Qing-Yu He, Jiake Xu, Langxia Liu

**Affiliations:** 1 Key laboratory of functional protein research of Guangdong higher education institutes, Institute of life and health engineering, Jinan University, Guangzhou, 510632, China; 2 School of Pathology and Laboratory Medicine, University of Western Australia, Perth, 6009, Western Australia, Australia

## Abstract

Osteoclast differentiation is a complex and finely regulated physiological process that involves a variety of signaling pathways and factors. Recent studies suggested that the Ser9 phosphorylation of Glycogen synthase kinase-3β (GSK3β) is required for the osteoclast differentiation. However, the precise underlying mechanism remains unclear. We have previously identified the heterogeneous nuclear ribonucleoprotein K (hnRNPK) as a putative GSK3β interactor. In the present study, we demonstrate that, during the RANKL-induced osteoclast differentiation, the PI3K/Akt-mediated Ser9 phosphorylation of GSK3β provokes the nuclear-cytoplasmic translocation of hnRNPK in an ERK-dependent manner, enhancing the cytoplasmic co-localization and interaction of GSK3β and hnRNPK. We show that hnRNPK is essential for the osteoclast differentiation, and is involved in several reported functions of GSK3β, including the activation of NF-κB, the expression of NFATc1, and the acetylation of tubulin, all known to be critical for osteoclast differentiation and functions. We find that hnRNPK is localized in the actin belt, and is important for the mature osteoclast formation. Taken together, we demonstrate here the critical role of hnRNPK in osteoclast differentiation, and depict a model in which the cytoplasmic hnRNPK interacts with GSK3β and regulates its function.

Originating from hematopoietic precursors of the monocyte-macrophage lineage, the osteoclasts are giant multinucleated cells whose excess number or super activity has been associated with several osteolytic diseases, such as osteoporosis, osteoarthritis, Paget’s disease of bone and giant cell tumor of bone^1^,^2^,^3^. Induced by cytokines such as M-CSF (Macrophage Colony-Stimulating Factor) and RANKL (Receptor activator of nuclear factor kappa-B ligand), the differentiation of the osteoclasts involves a variety of signal pathways and factors^4^,^5^. RANKL has been reported to activate a multitude of signaling pathways and to be required for the differentiation of osteoclasts. IKK (IκB kinase), NF-κB (nuclear factor κB), JNK (c-Jun N-terminal kinase), Akt, c-Src, p38, ERK (Extracellular signal-regulated kinases), AP-1 (activator protein 1), and NFATc1 (nuclear factor and activator of transcription) have all been shown to be the downstream effectors of RANKL during the osteoclast differentiation^6^,^7^,^8^.

Osteoclasts are characterized by the actin rich adhesive structure called podosomes that are important for the adhesion, motility, rigidity, topology, antigen sampling, and particularly the degradation of extracellular matrix of invasive cells^9^. Dynamic patterning of podosomes has been observed during the migration, fusion and bone resorption of the osteoclast. Podosomes can be organized in “patches”, “rosettes”, or transformed into a peripheral “actin belt” that is also referred as to “sealing zone” during the bone absorption. The actin belt has been shown to be required for the bone resorption activity and also for the fusion between the maturing osteoclasts^10^,^11^.

Glycogen synthase kinase-3β (GSK3β) is a multifunctional serine/threonine kinase capable of phosphorylating a wide range of substrates, and participate in a variety of cellular processes associated with the cell fate determination^12^,^13^. During the last years, several studies have pointed out the crucial role of GSK3β in the osteoclast differentiation^14^,^15^. The inactivation of GSK3β through its Ser9 phosphorylation has been shown to be required for the osteoclast differentiation^14^. The Ser9 phosphorylation of GSK3β by Akt has been associated with the downstream activation of NFATc1^15^, as well as the microtubule stabilization that is important for the actin belt formation^16^. However, the precise molecular mechanism underlying the functions of GSK3β remains to be investigated.

In a recent study, we have identified hnRNPK as one of the putative interacting partners of GSK3β in human Hepatocellular carcinoma HepG2 cells^17^. hnRNPK belongs to the DNA/RNA binding hnRNP (heterogeneous nuclear ribonucleoproteins) family and shares with several other RNPs the triple K-homology domain. hnRNPK has been found to shuttle between nucleus and cytoplasm, and its function has been so far mainly associated with RNA metabolism, including transcription, splicing, nuclear export, translation and decay^18^,^19^,^20^,^21^. However, due to its more specific KI domain (a.a. 240–337) which is a domain involved in the interactions with numerous kinases, hnRNPK might also be able to participate in the regulation of cell signaling^22^. In fact, hnRNPK contains within and around this domain multiple target sites for various kinase cascades, such as Y230/234/236 for Src/Lck/Lyn/Fyn, S284 for ERK, S302 for PKC, and S353 for JNK/ERK. It could therefore act as the effector of these kinases through the phosphorylation regulating its subcellular localization as well as its activity^22^,^23^,^24^,^25^,^26^,^27^,^28^,^29^. Particularly, its interaction with GSK3β might contribute to the regulation of the osteoclast differentiation. In the present study, we demonstrate the interaction between GSK3β and hnRNPK in mouse leukemic monocyte macrophage RAW264.7 cells, and address the functional significance of this interaction and its underlying mechanism in the RANKL-induced differentiation of osteoclast.

## Results

### Activation of PI3K/Akt pathway leads to Ser9 phosphorylation of GSK3β during the RANKL-induced osteoclast differentiation

**Figure 1:**
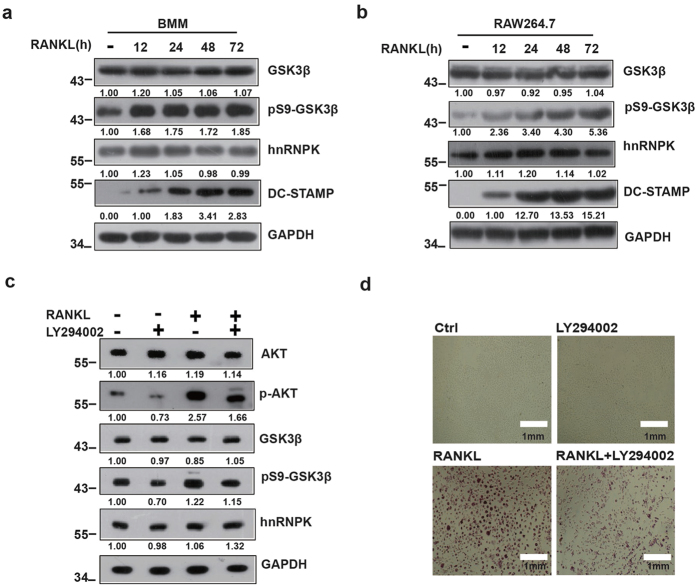
PI3K/AKT pathway promotes GSK3β Ser9 phosphorylation during the RANKL-induced Osteoclast differentiation. BMM or RAW264.7 cells were treated with RANKL for the indicated durations, then analyzed by Western blotting using the indicated antibodies (**a**,**b**). (**c**,**d**) BMM were treated with RANKL alone or in combination with PI3K inhibitor LY294002 then subjected to Western blotting using the indicated antibodies or TRAP staining as described in Materials and Methods. The numbers under each protein band in the blots indicate the fold change after normalization using GAPDH and in comparison with the control group (except for DC-STAMP for which the comparisons were made with the “12 hours” group because the protein amount was obviously 0 in the control group). Gels were run under the same experimental conditions. For better clarity and conciseness of the presentation, cropped blots are shown. The raw uncropped images can be found in the Supplementary Figure 1. The cropping lines are indicated by black lines both in cropped and uncropped images. The results shown are representative of at least 3 experiments.

We firstly examined the expression level of total GSK3β and hnRNPK, as well as the level of Ser9 phosphorylated GSK3β during the RANKL-induced differentiation of RAW264.7 cells and the primary mouse Bone Marrow derived Macrophages (BMM). Cells stimulated by 100 ng/mL RANKL for various durations were either subjected to Tartrate-resistant Acid Phosphatase (TRAP) activity staining, or collected and analyzed by Western blotting analysis using the appropriate antibodies. As expected, both types of cells could differentiate into mature multi-nucleated osteoclasts after the induction of RANKL for 48–72 hours (data not shown). During the entire period, no obvious variation of GSK3β or hnRNPK expression could be observed (Fig. 1a,b). However, consistent with previous reports, we observed a significant increased level of Ser9 phosphorylation of GSK3β during the differentiation of both types of cells. The RANKL-induced differentiation of both types of cells was also checked by the increased expression of DC-STAMP, a differentiation marker (Fig. 1a,b). The phosphorylation of GSK3β by activated Akt during the differentiation of osteoclast was then demonstrated (Fig. 1c). As shown, induction by RANKL resulted in the augmentation of phosphorylation level of Akt and GSK3β, while the PI3K inhibitor LY294002 reduced these effects of RANKL, confirmed by the result of TRAP activity staining for the corresponding effects on the differentiation of the cells (Fig. 1d). As expected, neither RANKL nor LY294002 affected the expression level of total GSK3β and hnRNPK proteins (Fig. 1c). Taken together, these results confirmed the serine 9 phosphorylation of GSK3β by PI3K/Akt during the RANKL-induced osteoclast differentiation, and its requirement for this cellular process.

### LiCl is unable to elicit the osteoclast differentiation but can enhance the effect of RANKL

**Figure 2:**
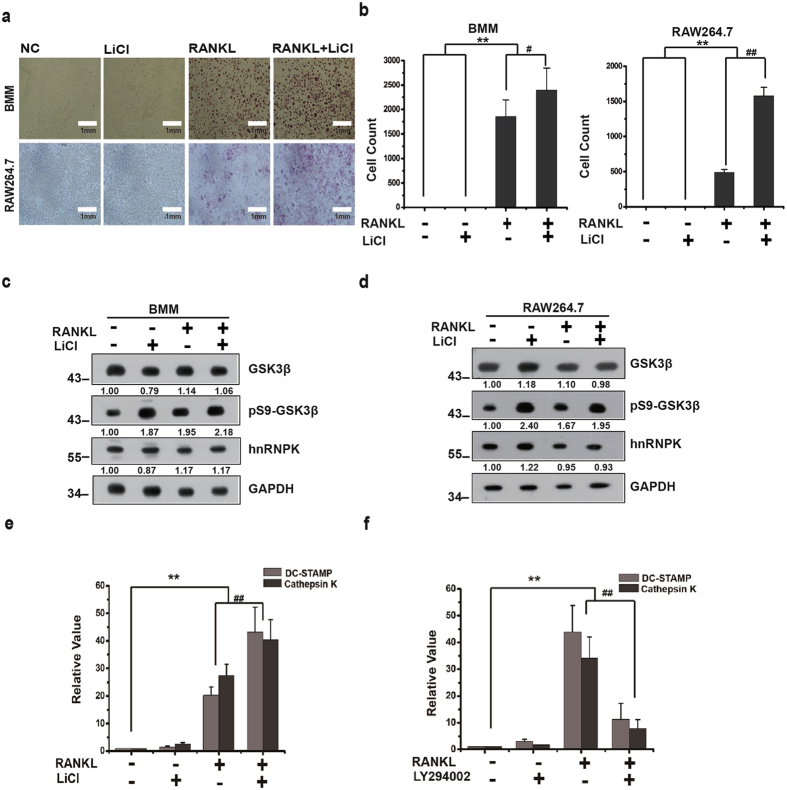
LiCl is unable to elicit the osteoclast differentiation but can enhance the effect of RANKL. (**a**) Primary BMM or RAW264.7 cells were treated separately or jointly with LiCl and RANKL, then subjected to TRAP staining. Cells were photographed under microscope. Scale bars = 1 mm (**b**) Quantification of TRAP-positive cells. The mean values of TRAP-positive cells in 5 randomly chosen fields are presented. **^/##^*P* < 0.01, ^#^*P* < 0.05. (**c**,**d**) Western blotting analysis of primary BMM or RAW264.7 cells treated as described in (**a**). The numbers under each protein band indicate the fold changes after normalization using GAPDH and in comparison with the control group. Gels were run under the same experimental conditions. For better clarity and conciseness of the presentation, cropped blots are shown. The raw uncropped images can be found in the Supplementary Figure 1. The cropping lines are indicated by black lines both in cropped and uncropped images. The results shown are representative of at least 3 experiments. (**e**,**f**) Total mRNA was extracted from RAW264.7 cells previously treated alone with RANKL, or in combination with either LiCl or LY294002, then reverse transcribed into cDNA. Real-time qPCR was subsequently performed for DC-STAMP and Cathepsin K expression. β-actin level was used for the normalization of the values. The results are presented as means ± SD of three independent experiments.

We further assessed the importance of Ser9 phosphorylation of GSK3β in the osteoclast differentiation by testing the effect of LiCl alone or in combination with RANKL on the differentiation of RAW264.7 cell and BMM. Cells treated separately or jointly by LiCl and RANKL were examined by Western blotting for the Ser9 phosphorylation level of GSK3β and for osteoclast differentiation by TRAP activity staining. We found that LiCl was unable to induce the differentiation of RAW264.7 or BMM cells in the absence of RANKL stimulation (Fig. 2a,b), although it could increase the phosphorylation level of GSK3β, regardless cells were treated with RANKL or not (Fig. 2c,d). However, a significant enhancement of differentiation could be observed in both RAW264.7 and BMM cells treated jointly by RANKL and LiCl, as compared with those treated by RANKL alone (Fig. 2a,b). These results were further confirmed using real-time PCR assays to detect the expression level of DC-STAMP (dendritic cell-specific transmembrane) and Cathepsin K, both specific markers of the osteoclast differentiation. RAW264.7 cells were treated either with RANKL or LiCl alone, or with RANKL combined with LiCl or the PI3K inhibitor LY294002. Total RNA was then extracted from these cells and subjected to reverse transcription and real-time quantitative PCR to assess the mRNA expression level of DC-STAMP and Cathepsin K, normalized over β-actin expression level. The results in Fig. 2e,f show that DC-STAMP and Cathesin K expressions were dramatically stimulated by RANKL but not by LiCl alone. And, as expected, LiCl enhances the effect of RANKL whereas LY294002 inhibits this effect. These results suggest that the Ser9 phosphorylation of GSK3β is required but not sufficient to elicit the osteoclast differentiation.

### RANKL or LiCl treatment induces the nuclear-cytoplasmic translocation of hnRNPK and enhances its interaction and co-localization with GSK3β

**Figure 3:**
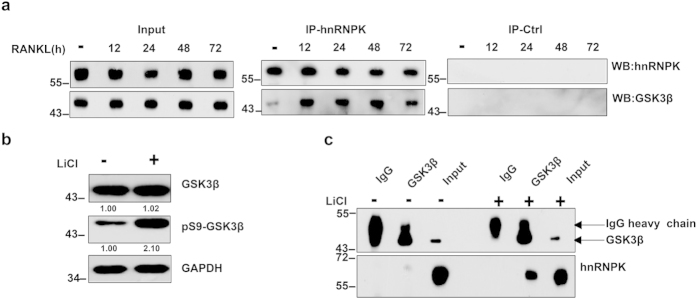
RANKL or LiCl enhances GSK3β-hnRNPK interaction. (**a**) Co-immunoprecipitations of hnRNPK and GSK3β using an anti-hnRNPK antibody (IP-K) performed with RAW264.7 cells treated with RANKL for the indicated durations, revealed by Western blotting with appropriate antibodies. Same co-IP experiments were also performed using non-immune mouse IgG as negative control (IP-Ctrl). Western blotting reveals the increased amount of GSK3β co-immunoprrecipitated with hnRNPK under RANKL induction. The total amounts of hnRNPK and GSK3β in the whole cell lysate were set as internal control. (**b**) RAW264.7 cells were treated or not with 10 mM LiCl during 8 hours, then harvested and lysed. Total and Ser9 phosphorylated GSK3β levels were assessed by Western blotting using appropriate antibodies. (**c**) Immunoprecipitation experiments using anti-GSK3β antibody were carried out with cell protein extracts of the cells treated as described in (**b**). GSK3β and hnRNPK in the immunoprecipitates were revealed by Western blotting using the corresponding antibodies. Non-immune IgG (IgG) was used as a negative control for the specific anti-GSK3β antibody. The numbers under each protein band in the blots indicate the fold change after normalization using GAPDH and in comparison with the control group. Gels were run under the same experimental conditions. For better clarity and conciseness of the presentation, cropped blots are shown. The raw uncropped images can be found in the Supplementary Figure 1. The cropping lines are indicated by black lines both in cropped and uncropped images. The results shown are representative of at least 3 experiments.

Following the identification of hnRNPK as a putative interacting partner of GSK3β in HepG2 cells, we have carried out co-immunoprecipitation experiment to confirm the interaction between these two proteins in RAW264.7 cells using respectively the anti-hnRNPK and anti-GSK3β antibodies. Curiously, in both cases, only faint binding of these two proteins could be detected, as judged by the very small amount of each interactor co-immunoprecipitated with the antibody of its counterpart (data not shown). Therefore, we investigated if the interaction between hnRNPK and GSK3β in RAW 264.7 cells might be regulated during RANKL-induced differentiation. To this end, co-immunoprecipitation experiments were again performed for hnRNPK and GSK3β with protein extracts of RAW264.7 cells treated by RANKL for various durations as described above. Interestingly, we observed a significant enhancement of GSK3-hnRNPK interaction upon RANKL treatment. This stimulation was maintained through the entire period of observation (Fig. 3a). We then asked if the enhancement of GSK3β-hnRNPK interaction might be related to the increased Ser9 phosphorylation of GSK3β, also induced by the RANKL treatment. To answer this question, we used LiCl to stimulate the serine 9 phosphorylation of GSK3β in RAW264.7 cells and examined its effect on GSK3β-hnRNPK interaction by co-immunoprecipitation assays. As expected, LiCl stimulated the serine 9 phosphorylation of GSK3β in cells while the total expression level of GSK3β remained unchanged (Fig. 3b). Interestingly, the treatment of RAW264.7 cells by LiCl dramatically increased the interaction between hnRNPK and GSK3β (Fig. 3c).

**Figure 4:**
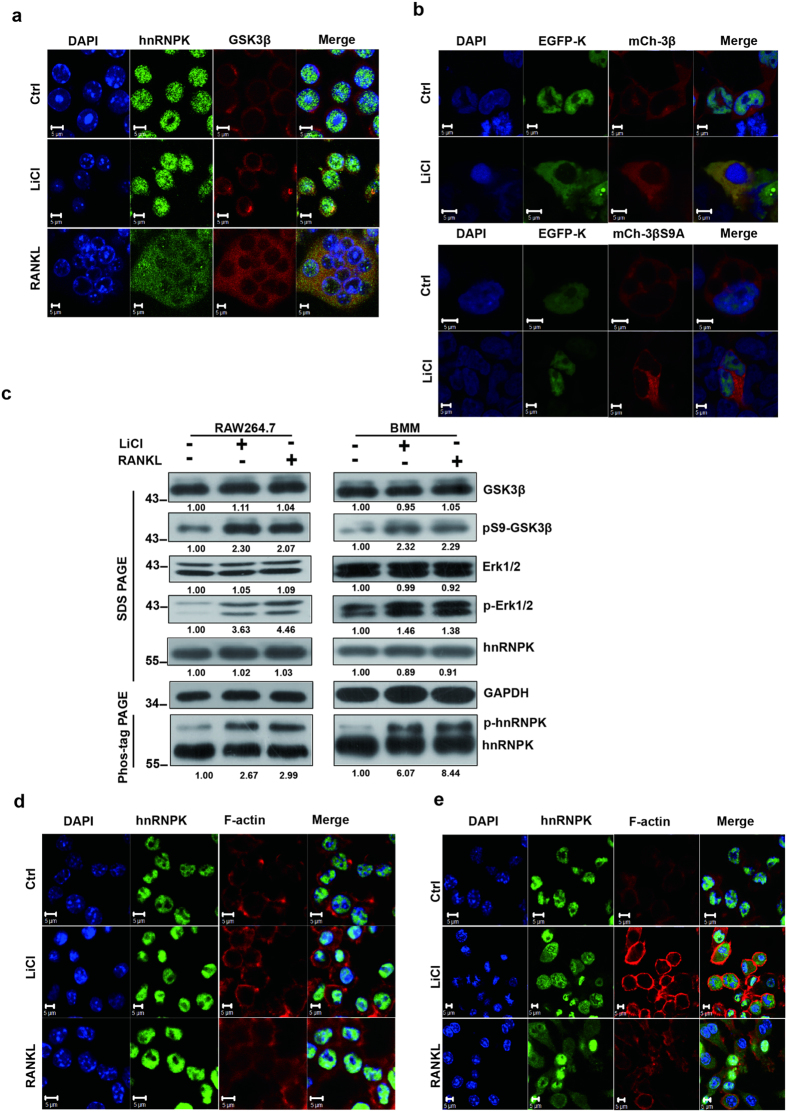
RANKL or LiCl induces the nuclear-cytoplasmic translocation of hnRNPK and its co-localization with GSK3β. (**a**) The subcellular distribution of hnRNPK and GSK3β by confocal immunofluorescence experiments with RAW264.7 cells treated with 10 mM LiCl during 12 hours, or with 100 ng RANKL during 72 hours. hnRNPK was stained in green and GSK3β in red. (**b**) HEK293T cells were co-transfected with EGFP-hnRNPK (EGFP-K) and mCherry-GSK3β *wt* (mCh-3β, upper panels) or mCherry-GSK3β S9A (mCh-3βS9, lower panels) constructs. The subcellular localization of these fusion proteins were then examined under confocal microscopy. Scale bars = 5 μM. (**c**) RAW264.7 or BMM cells were treated either with 10 mM LiCL or 100 ng/mL RANKL during 12 hours, the protein extract of these cells were subjected to Western blotting to assess the total and phosphorylated levels for ERK or GSK3β, the phosphorylated and unphosphorylated levels of hnRNPK using appropriate antibodies. The numbers under each protein band indicate the fold changes after normalization using GAPDH and in comparison with the control group. For hnRNPK, only the quantitation result of the phosphorylated form was presented. Gels were run under the same experimental conditions except for the Phos-tag-PAGE. For better clarity and conciseness of the presentation, cropped blots are shown. The raw uncropped images can be found in the Supplementary Figure 1. The cropping lines are indicated by black lines both in cropped and uncropped images. The results shown are representative of at least 3 experiments. (**d**) RAW264.7 cells were co-treated by either 100 ng/mL RANKL for 48 hours or 10 mM LiCl for 12 hours with 20 μM u0126, and subjected to immunofluorescence assays to determine the subcellular localization of hnRNPK. (**e**) In parallel, control cells were co-treated by either 100 ng/mL RANKL for 48 hours or 10 mM LiCl for 12 hours with DMSO, and analyzed similarly as in (**d**). The results shown are representative of at least 3 experiments.

To further confirm the interaction of GSK3β and hnRNPK during the RANKL-induced osteoclast differentiation and its possible regulation by the serine 9 phosphorylation of GSK3β, we performed confocal microscopy experiments to examine the sub-cellular distribution of GSK3β and hnRNPK in RAW264.7 cells under RANKL or LiCl treatment, and in non-treated RAW264.7 cells. Interestingly, we found that in non-treated control cells, GSK3β was mainly located in the cytoplasm while hnRNPK was exclusively in the nucleus (Fig. 4a, upper panels). However, upon either LiCl or RANKL treatment, there was an obvious translocation of hnRNPK to the cytoplasm, resulting in an enhancement of co-localization of GSK3β and hnRNPK in the cytoplasm (Fig. 4a, middle and lower panels).

In order to further investigate the causal relationship between the Ser9 phosphorylation of GSK3β and the nuclear-cytoplasmic translocation of hnRNPK, EGFP-hnRNPK construct with either wild type mCherry-GSK3β or the phosphorylation-defective mCherry-GSK3β S9A mutant was co-transfected into HEK293 cells, and the subcellular localization of these proteins was again examined by confocal microscopy. As shown in Fig. 4b, when co-transfected with mCherry-GSK3β *wt*, EGFP-hnRNPK displayed an expected nuclear-cytoplasmic translocation upon LiCl treatment (upper panels). However, this translocation did not occur when it was co-transfected with mCherry-GSK3β S9A (lower panels). This result demonstrates clearly that the translocation observed was indeed associated with the serine 9 phosphorylation of GSK3β, but not with any other consequence of LiCl treatment. It has been previously reported that LiCl decreased the ERK-GSK3β interaction in the cytoplasm and translocated ERK to the nucleus^30^. Another study has demonstrated that ERK kinase phosphorylated hnRNPK at its serines 284 and 353 and caused its cytoplasmic accumulation^24^. Therefore, we investigated if the translocation of hnRNPK induced by LiCl or RANKL described above was mediated by ERK activity. To this end, we firstly examined the phosphorylation state of both ERK and hnRNPK under the effect of LiCL and RANKL. RAW264.7 or BMM cells were treated either with 10 mM LiCL or 100 ng/mL RANKL during 12 hours, the protein extract of these cells were subjected to Western blotting to assess the total (or unphosphorylated), and phosphorylated levels for ERK and hnRNPK. Because we did not dispose an antibody against the phosphorylated form of hnRNPK, we took advantage of the Phos-tag PAGE system to visualize both the phosphorylated and unphosphorylated hnRNPK using an anti-hnRNPK antibody. As shown in Fig. 4c, In both RAW264.7 (left panels) and BMM (right panels) cells, the treatment of LiCl or RANKL indeed led to the increase of phosphorylation level of ERK and also hnRNPK, as well as the expected increase of Ser9 phosphorylation of GSK3β. To go farther from this result, RAW264.7 cells were treated with the ERK inhibitor u0126, and examined by confocal immunofluorescence the effect of LiCl or RANKL on the sub-cellular distribution of hnRNPK. As shown in Fig. 4d (cells with u126) and e (control cells with DMSO), the inhibition of ERK abrogated the nuclear-cytoplasmic translocation induced by LiCl or RANKL.

### hnRNPK is essential for the RANKL-induced osteoclast differentiation

**Figure 5:**
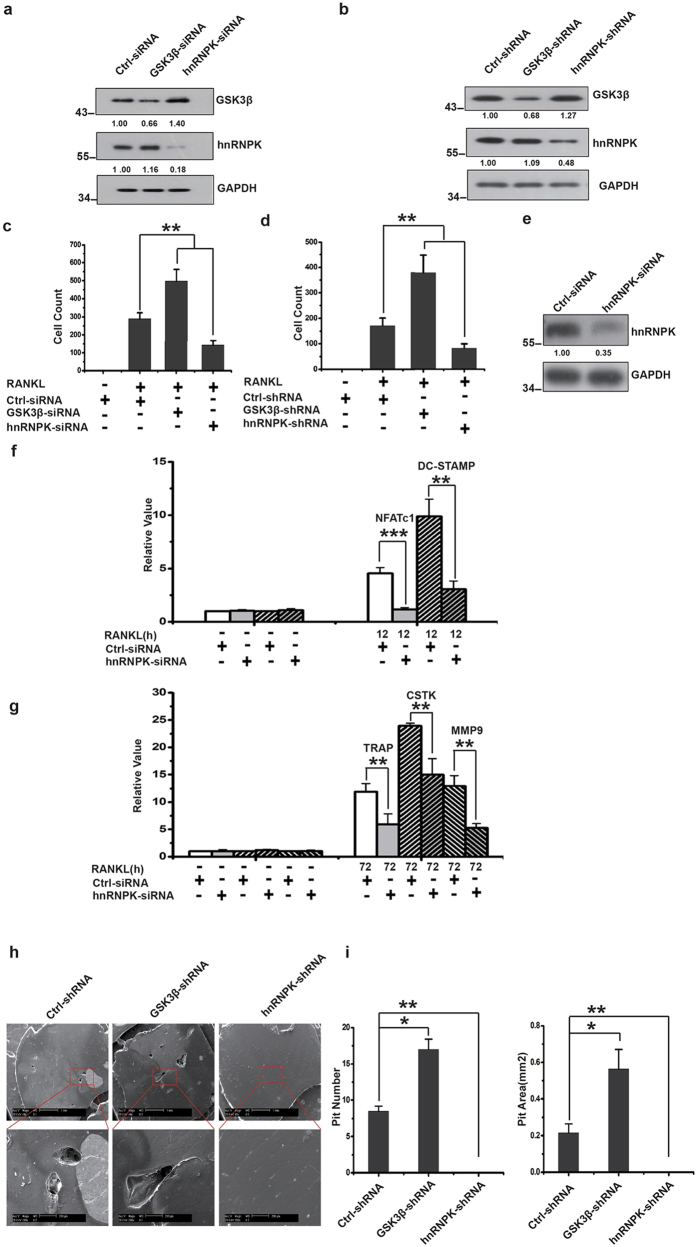
hnRNPK is essential for the RANKL-induced osteoclast differentiation. (**a**) Western blotting showing the effect of hnRNPK or GSK3β knockdown in RAW264.7 cells 24 hours after siRNA transfection. The numbers under each protein band indicate the fold changes after normalization using GAPDH and in comparison with the control group. (**b**) hnRNPK or GSK3β was knocked down using lentiviral-mediated shRNA in primary BMM. Western blotting with cell lysates was performed 48 hours after viral infection. The results shown are representative of at least 3 experiments. Quantification of TRAP-positive cells with (**c**) RAW264.7 cells, or (**d**) BMM performed 72 hours after RANKL induction to assess the degree of differentiation. Cells were photographed under microscope. TRAP-positive cells in 5 randomly chosen fields were counted. **^/##^*P* < 0.01. The results presented were the means ± SD of three independent experiments. (**e**) Western blotting showing the **e**ffect of hnRNPK knockdown in the cells used in (**f**,**g**). (**f**,**g**) RAW264.7 cells were transfected by hnRNPK siRNA, then induced with 100 ng/mL RANKL. At the time points of 0 (“-”, before induction), 12 and 72 hours, total RNA were extracted, reverse-transcribed into cDNA, and the indicated specific mRNA were quantified by real-time PCR using appropriate primers. ***P < 0.001. **P < 0.01. The results presented were the means ± SD of three independent experiments. The number under each protein band indicate the fold changes after normalization using GAPDH and in comparison with the control group. (**h**) hnRNPK or GSK3β was knocked down using lentiviral-mediated shRNA in primary BMM. *In vitro* bone-resorption assays were then performed as described. The scanning electron micrographs shown are representative of the bone slices for the indicated experimental groups. Cells infected by lentiviruses bearing non-target control shRNA were used as control. Scale bars represent 1 mm for the upper panels and 200 μm for the lower panels. (**i**) Quantification of the resorption pits. Resorption p**i**ts on each bone slice were counted. Pit areas were recognized and calculated with the help of Adobe Photoshop CS5. The sum of the pit number (left) and the pit area (right) on a bone slice are shown. The results presented are the means ± SD of three independent experiments. **^/##^*P* < 0.01.

The nuclear-cytoplasmic translocation of hnRNPK, together with its enhanced interaction with GSK3β during the RANKL-induced osteoclast differentiation suggests that hnRNPK might play an important role in this cellular process. To answer this question, hnRNPK or GSK3β was knocked down by using specific siRNA in RAW264.7 cells (Fig. 5a), and by using the corresponding lentiviral-mediated shRNA in BMM cells (Fig. 5b). The effect of these treatments on the RANKL-induced differentiation was then tested by TRAP activity staining. The quantification of the TRAP-positive cells gave very similar results for both types of cells (Fig. 5c,d). Interestingly, we found that the knockdown of hnRNPK significantly inhibited the differentiation in both types of cells, suggesting that this protein is essential for the differentiation of osteoclast. In contrast, the knockdown of GSK3β dramatically stimulates the differentiation of RAW264.7 cells and BMM, which is consistent with previous reports by others^14^,^15^. The critical role of hnRNPK in the osteoclast differentiation was then confirmed by real-time quantitative PCR assessing the mRNA level of various differentiation specific markers during the RANKL-induced differentiation in hnRNPK knocked down RAW264.7 cells. As shown in Fig. 5e–g, hnRNPK knockdown affects the expression of the differentiation markers expressed at the early stage (12 hours post RANKL induction), such as NFATc1 and DC-STAMP, as well as those expressed at the late stage of differentiation (72 hours post RANKL induction), such as CTSK (Cathepsin K), TRAP and MMP9 (Matrix Metalloproteinase 9).

In order to further confirm the critical importance of hnRNPK in the osteoclast differentiation by a functional assay, we evaluated the *in vitro* bone resorbing ability of both hnRNPK-knocked down and GSK3β-knocked down BMM-derived osteoclasts. BMM were stimulated with M-CSF and RANKL, and subsequently infected by lentiviruses carrying the appropriate shRNA and cultured under G418 antibiotic for selection. The selected cells were then seeded on the cow bone slices to test their ability of forming the absorbing pits as described in Experimental Procedures. Consistently, cells treated with hnRNPK shRNA completely lost the ability of bone resorption, whereas GSK3β shRNA enhanced this ability (Fig. 5h,i). These results further confirmed the involvement of hnRNPK in the osteoclast differentiation.

### GSK3β Ser9 phosphorylation and hnRNPK are both involved in the NF-κB activation and NFTAc1 expression during the osteoclast differentiation

**Figure 6:**
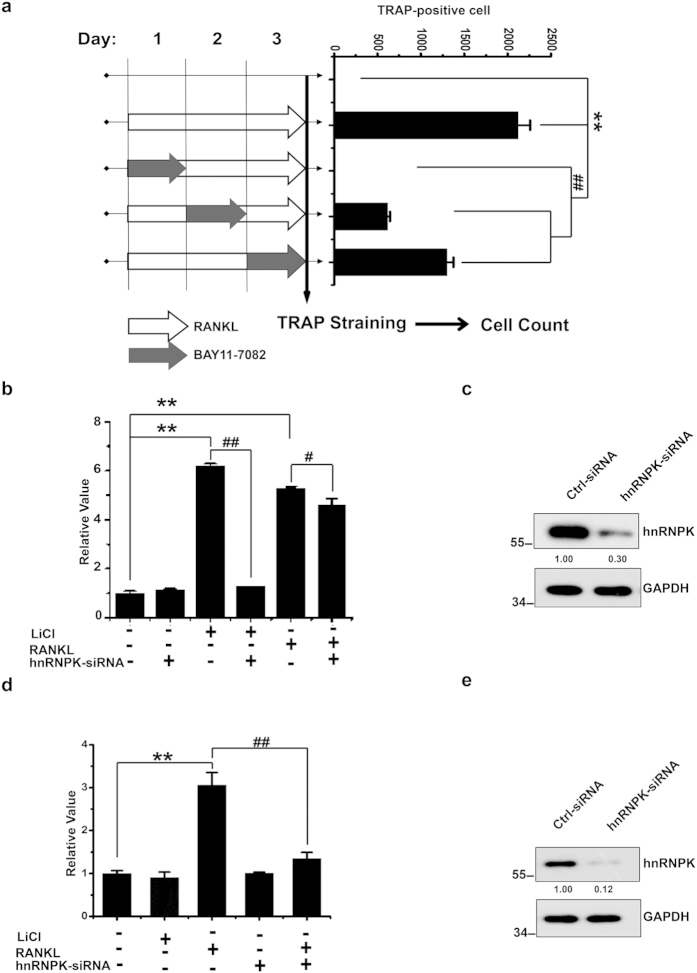
GSK3β Ser9 phosphorylation and hnRNPK are both required for the NF-κB activation and NFTAc1 expression during the osteoclast differentiation. (**a**) RAW264.7 cells were co-treated with NF-κB inhibitor BAY 11-7082 respectively during the first, second, and third day of RANKL treatment. TRAP staining was performed after 3 days of RANKL treatment, and the total TRAP-positive cells in each well of the 96-well microplate were counted. The data presented are the means ± SD of three wells. **^/##^*P* < 0.01. (**b**) Luciferase activity assays with RAW-NF-κB cells under the indicated treatments as described in material and methods section. The results were expressed as the folds of the value of non-treated control group and presented are the means ± SD of four independent experiments. **^/##^*P* < 0.01, ^#^*P* < 0.05. (**c**) Western blotting to check the knockdown of hnRNPK in the cells used in (**b**). (**d**) Assessment of NFATc-1 mRNA level in RAW264.7 cells by real-time qPCR after the indicated treatments. The results were expressed as the folds of the value of non-treated control group and presented are the means ± SD of three independent experiments. (**e**) Western blotting demonstrating the effect of hnRNPK knockdown in the cells described in (**d**). Th**e** numbers under each protein band indicate the fold changes after normalization using GAPDH and in comparison with the control group. The results shown are representative of at least 3 experiments.

NF-κB pathway has been demonstrated by several studies to be critical for osteoclast survival, differentiation and bone resorbing activity^31^,^32^,^33^,^34^,^35^,^36^,^37^,^38^,^39^. Furthermore, it has also been showed that GSK3β is an important regulator of NF-κB pathway^40^,^41^,^42^,^43^,^44^,^45^. In order to confirm the importance of NF-κB pathway in RANKL-induced osteoclast differentiation, we have treated the RAW264.7 cells respectively during the first day, the second day or the third day of RANKL induction with the inhibitor of NF-κB pathway BAY 11–7082, and assess the impact on the differentiation of these cells by TRAP staining after three days of RANKL induction. Our results showed that all cells treated by BAY 11–7082 were affected in their differentiation, regardless the different timing of treatment (Fig. 6a). Furthermore, earlier the cells were treated, greater was the effect. We observed above all a drastic decrease of the total cell numbers, confirming that the activation of NF-κB pathway is important in the early stage of the osteoclast differentiation, probably by playing a critical role in the survival of the maturing osteoclasts. We then examined the role of GSK3β-hnRNPK interaction in the activation of NF-κB by using a Luciferase reporter system with a RAW264.7 cell line stably expressing the firefly Luciferase reporter gene under the control of NF-κB (referred as to RAW-NF-κB). We found that LiCl or RANKL could lead to a 6 folds increase of NF-κB activity, whereas hnRNPK knockdown exerted no significant effect on the NF-κB activity in non-treated cells. However, interestingly, the stimulating effect of LiCl treatment was totally abrogated by hnRNPK knockdown (Fig. 6b,c).These results suggest that both the Ser9 phosphorylated GSK3β and the normal expression of hnRNPK are required for the NF-κB activation in RAW264.7 cells, and their functions are interdependent of each other since one without the other resulted in the defect of NF-κB activation. Curiously, hnRNPK knockdown exerted only a faint effect on NF-κB activation induced by RANKL, hinting on the existence of alternative NF-κB pathway independent of GSK3β and hnRNPK involvements (Fig. 6c).

NFATc1 is considered to be the master transcriptional factor for osteoclast differentiation due to its ability to regulate the expression of numerous osteoclast-specific genes^5^,^46^,^47^,^48^,^49^. Meanwhile, Ser9 phosphorylation of GSK3β has been recently involved in the NFATc1 activation during the osteoclast differentiation^14^,^15^. Therefore, we investigated the putative role of GSK3β-hnRNPK interaction in the NFATc1 expression. Firstly, real-time quantitative PCR assays have been performed to assess the level of NFATc1 mRNA expression in RAW264.7 cells with the indicated treatments (Fig. 6d,e). As shown, LiCl alone had no effect on NFATc1 expression even if it was capable of stimulating the activity of NF-κB as described above. This result is in line with the previous reports that both NF-κB and c-Fos pathways activated by RANKL are required for the activation of NFATc1^49^, and confirm also our observation that LiCl alone is unable to induce osteoclast differentiation. As expected, RANKL significantly stimulated the expression level of NFATc1, and more interestingly, this effect was severely reduced by hnRNPK knockdown (up to 83% of reduction, Fig. 6d). Our result suggests that hnRNPK promotes the expression of NFATc1 in RANKL-induced osteoclasts.

### hnRNPK is important for the acetylation of tubulin and the formation of mature multinucleated osteoclast

**Figure 7:**
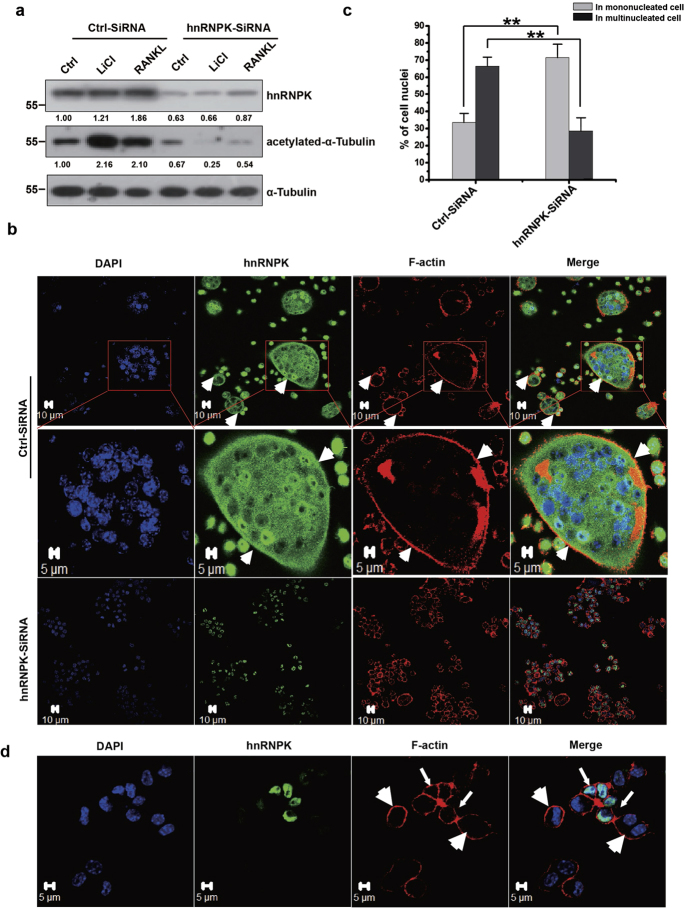
hnRNPK is required for the acetylation of tubulin and the formation of mature multinucleated osteoclast. (**a**) The effect of hnRNPK knockdown on the tubulin acetylation. RAW264.7 cells were transfected by hnRNPK or control siRNA, and 24 hours later, stimulated by 100 ng/mL RANKL or 10 mM LiCl for 12 hours. Western blotting was then performed using the indicated antibodies. The numbers under each protein band indicate the fold changes after normalization using α-tubulin and in comparison with the control group. Gels were run under the same experimental conditions. For better clarity and conciseness of the presentation, cropped blots are shown. The raw uncropped images can be found in the Supplementary Figure 1. The cropping lines are indicated by black lines both in cropped and uncropped images. The result shown is representative of three independent experiments. (**b**) The effect of hnRNPK knockdown on the formation of the multi-nucleated osteoclasts. RAW264.7 cells were transfected by hnRNPK siRNA or control siRNA, and 24 hours later, stimulated by 100 ng/mL RANKL for 72 hours. Confocal immunofluorescence was performed as indicated. hnRNPK is stained in green, F-actin is stained in red with phalloidin, and nuclei are stained in blue with DAPI. Arrow heads indicate the co-localization of hnRNPK and F-actin in the periphery of the cell. Scale bars = 5 μM/10μM as indicated. The result shown is representative of three independent experiments. (**c**) Quantification of the cell nuclei in the mono- or multi-nucleated cells (>=3 nuclei) described in (**b**). The results presented are the mean values obtained from 5 randomly chosen fields. ***P < 0.01. (**d**) hnRNPK knockdown has no effect on the actin belt formation. RAW264.7 cells were transfected by hnRNPK siRNA, and 24 hours later, stimulated by 100 ng/mL RANKL for 24 hours. Confocal immunofluorescence experiment was then performed to visualize the actin belt formation and the effect of hnRNPK knockdown by siRNA. Cells in which hnRNPK has been successfully depleted are indicated by the big arrow heads whereas those still express hnRNPK are indicated by the small arrows. Scale bars = 5 μM. The result shown is representative of three independent experiments.

It has been previously reported that the formation of the actin belt in osteoclasts depends on the microtubule dynamic instability, and is regulated by the post-transcriptional modifications of tubulin^50^,^51^,^52^,^53^. Particularly, the acetylated tubulin has been observed in the peripheral cytoplasm and associated with actin belt in mature osteoclast^45^,^46^. Furthermore, studies by Matsumoto and colleagues have demonstrated that Akt/GSK3β pathway affected the actin belt (sealing zone) formation and the bone resorbing ability of the osteoclast through regulating the acetylation of tubulin and the microtubule stability^16^. Therefore, we investigated if hnRNPK might also, through interaction with GSK3β, be involved in the acetylation of tubulin and the formation of actin belt in the RANKL-induced osteoclasts. Firstly, RAW264.7 cells were transfected by hnRNPK siRNA or control siRNA, treated with RANKL or LiCl as described, and examined by Western blotting for the expression levels of hnRNPK, α-tubulin and the acetylated α-tubulin. Interestingly, we observed that LiCl or RANKL treatment significantly enhanced the acetylation of α-tubulin, and this stimulatory effect was dramatically reduced by hnRNPK knockdown (Fig. 7a). Confocal immunofluorescence experiments were then performed to visualize the pattern of podosomes in RANKL-induced RAW264.7 cells transfected by hnRNPK siRNA or control siRNA. Interestingly, we found that hnRNPK knockdown resulted in a significant reduction in percentage of the multi-nucleated cells (with at least 3 nuclei) with actin belt (Fig. 7b,c). Moreover, hnRNPK displayed a clear co-localization with F-actin in the periphery of the mature multi-nucleated osteoclasts. It was localized within, and also adjacent to the actin belt (Fig 7b, indicated by arrow heads), suggesting that hnRNPK might be associated with actin belt, as it has been reported for tubulin^44^,^45^. This result is reminiscent of a proteomic study which has identified hnRNPK in the podosome-enriched fraction of primary macrophages^54^. In our experiments, we have not been able to observe any obvious effect of hnRNPK knockdown on the actin belt formation (Fig. 7d). These results suggest that hnRNPK might, by regulating the tubulin acetylation through interaction with GSK3β, play a role in the fusion between the maturing osteoclasts to form the multi-nucleated mature osteoclasts, rather than in the formation of actin belt.

## Discussion

The Ser9 phosphorylation of GSK3β, associated with its loss of Ser/Thr kinase activity, has been reported to be determinant for its multiple physiological functions^12^. Particularly, it has recently been shown to be required for the osteoclast differentiation. While the simple GSK3β knockdown or knockout promoted the osteoclast differentiation, it is nonetheless interesting to note that RANKL induces the Ser9 phosphorylation of GSK3β but not the down regulation of its total protein level during the differentiation of osteoclast^14^,^15^. This might suggest that extremely fine and dynamic regulation of GSK3β activity associated with its Ser9 phosphorylation is important for the osteoclast differentiation and function. Interestingly, in the present study, we found that during RANKL-induced osteoclast differentiation, Ser9 phosphorylation of GSK3β may trigger the nuclear-cytoplasmic translocation of hnRNPK, stimulating its interaction with GSK3β. Moreover, our studies suggest that the function of the Ser9 phosphorylated GSK3β is dependent on hnRNPK for the differentiation and bone resorbing activity of the osteoclast. Thus, hnRNPK, whose interaction with GSK3β and whose function during the osteoclast differentiation correlate tightly with the Ser9 phosphorylation of GSK3β, may play an important role in the regulation of the activity of the latter. Moreover, the Ser9 phosphorylation of GSK3β is generally considered to be a “loss of function” modification due to its inhibiting effect on the kinase activity of GSK3β^12^. But it is not excluded that, by Ser9 phosphorylation, GSK3β might also gain novel functions unrelated to its Ser/Thr kinase activity, for example, interacting with other proteins and regulating their activities in one way or another. Therefore, the investigation on the physical and functional interactions between these two proteins would be important for a better understanding of GSK3β functions in the osteoclast differentiation, as well as in other cellular processes determining the cell fate such as the cell apoptosis, in which GSK3β plays a very important role.

Due to the versatile nature of GSK3β, its functions in the osteoclast differentiation might be achieved via multiple mechanisms that might impact on different molecular events. So far, several studies have suggested its role in the regulation of NFATc1 activity and the acetylation of tubulin^14^,^15^,^16^. We have been able to demonstrate the possible involvement of hnRNPK in these two functions of GSK3β, as well as its involvement in the regulation of NF-κB pathway by GSK3β. Previous studies suggested that NF-κB pathway might regulate the expression of NFTAc1 in the osteoclast. In our study, the Ser9 phosphorylation of GSK3β in RAW264.7 cells induced by LiCl treatment activates the NF-κB pathway while it was not sufficient for the up-regulation of NFTAc1 expression. However, hnRNPK knockdown resulted in the defection of both the activation of NF-κB induced by RANKL or LiCl, and that of the NFTAc1 expression induced by RANKL. This might suggest that, in the context of RANKL induction, the interaction between Ser9 phosphorylated GSK3β and hnRNPK is required for the activation of NF-κB pathway which, in cooperation with other signaling pathways activated by RANKL induction, stimulates the expression of NFTAc1. How the interaction of Ser9 phosphorylated GSK3β and hnRNPK could regulate the NF-kB pathway during the osteoclast differentiation needs further investigations in the future, since different and somehow confusing results about the activation and regulation of several sub-pathways (including the canonical and non-canonical NF-κB pathways, and the “RANKL-independent” or “IKK-independent” pathways) have been reported by the literature^31^,^32^,^33^,^34^,^35^,^36^,^37^,^55^.

Based on the known activities of both hnRNPK and GSK3β reported in the literature and the finding in our study, we believe that hnRNPK may intervene at multiple stages and molecular events during the osteoclast differentiation. On one hand, our results showing the prompt up-regulation of the Ser9 phosphorylation of GSK3β upon RANKL induction, the involvement of hnRNPK-GSK3β interaction in NF-κB activation, and also the hnRNPK-dependent up-regulation of the early differentiation markers suggest that hnRNPK plays a critical role from the early stage of differentiation (Figs 1,5 and 6). On the other hand, the interesting role of hnRNPK in the regulation of tubulin acetylation indicates that it may also be important for the late stage of differentiation and function of the osteoclasts (Fig. 7). It has been since longtime reported that the cytoskeletons, including the microtubules, are important for NF-κB activation^56^,^57^,^58^,^59^. As described hereinabove, we have shown that both RANKL and LiCl enhanced the acetylation of tubulin in an hnRNPK-dependent manner (Fig. 7). Therefore, it could be possible that the hnRNPK-dependent activation of NF-κB pathway by RANKL and LiCl could be mediated by the microtubule network through the regulation of tubulin acetylation. However, the microtubule instability regulating the formation and dynamic patterning of the podosomes is also involved in the late differentiation processes and functions of the osteoclasts, such as the cell movement, fusion and bone resorption^50^,^52^,^53^,^60^. Therefore, the importance of hnRNPK in the late stage of differentiation seems probable even though it remains to be proved.

We have been able to show in our bone resorption assays that hnRNPK knockdown resulted in the loss of resorptive ability of osteoclasts. However, this could not allow us to draw any conclusion about the direct involvement of this protein in the bone resorption process of the cell, due to the devastating effect of its knockdown on the formation of mature osteoclasts. In this case, the loss of resorbing activity should simply reflect the differentiation defect of these cells. Future studies by using the appropriate technical approaches, such as an inducible knockout/knockdown system, may permit to directly demonstrate the involvement of hnRNPK in the fusion as well as the resorption activity of the osteoclast.

**Figure 8:**
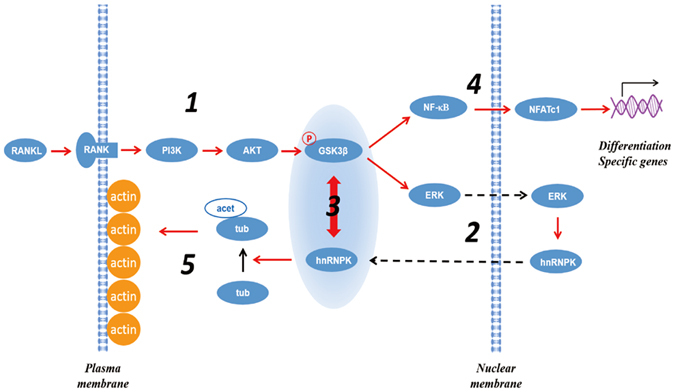
Cytoplasmic hnRNPK interacts with GSK3β and is essential for the osteoclast differentiation. The activation of PI3K/Akt pathway by RANKL stimulates the Ser9 phosphorylation of GSK3β (1), which in turn induces the nuclear-cytoplamic translocation of hnRNPK mediated by ERK activity (2), and thereby enhances GSK3β-hnRNPK interaction (3). This interaction, on one hand, is required for the activation of NF-κB pathway and the expression of NFATc-1, which results in the expression of the osteoclast specific genes (4), and on the other hand, is involved in the acetylation of α-tubulin that is important for the fusion between the maturing osteoclasts and the resorbing ability of the mature osteoclasts (5).

In conclusion, the GSK3β-hnRNPK interaction might intervene at multiple steps or molecular processes in the osteoclast differentiation, probably through different molecular mechanisms (Fig. 8). Further clarification of each of these molecular mechanisms will be the aim of our upcoming studies.

## Materials and Methods

### Cells, Plasmids and Reagents

RAW264.7 cells was from ATCC, and RAW264.7-NFκB cells were obtained by stable transfection of pNFκB-TA-Luc plasmid (Clontech) into RAW264.7 cells as previously described^61^. Monoclonal GSK3β antibody (27C10) and monoclonal pSer9-GSK3β antibody were purchased form Cell Signaling Technology. Mouse monoclonal hnRNPK antibody (D-6) was from Santa Cruz Biotechnology (Santa Cruz, CA). Rabbit polyclonal hnRNPK antibody, α- and β- tubulin antibodies were from Proteintech Group, Inc. (Chicago, USA). Acetylated α-tubulin antibody was from Sigma-Aldrich (St. Louis, MO). ERK (#9102) and phosphorylated ERK (20G11, #4376) Rabbit mAb were from Cell Signaling Technology (CST). DC-STAMP antibody (H-91) was from Santa Cruz. Monoclonal GAPDH antibody was obtained from ZSGB Biotechnology. Secondary antibodies used in confocal were from Jackson ImmunoResearch (West Grove, PA). hnRNPK and GSK3β siRNAs were purchased from GenePharma. Lentiviruses bearing hnRNPK and GSK3β shRNAs were purchased from GeneChem (China). Alpha modified of Eagles Medium (α-MEM) and fetal bovine serum were purchased from Gibco Life Technologies. PI3K inhibitor LY294002, NFκB inhibitor BAY 11-7082 and ERK inhibitor u0126 were purchased from Beyotime Institute of Biotechnology (Nantong, Jiangsu, China). Phos-tag reagent was purchased from Wako Pure Chemical Industries, Ltd, Japan. The pGEX-6P-1 expression vector was used to express RANKL^62^.

### Cell Culture and transfection

RAW264.7 cells and RAW264.7-NFκB cells were cultured in α-MEM supplemented with 10% fetal bovine serum (FBS). Cells were incubated at 37 °C in a humidified atmosphere of 5% CO2. RAW264.7-NFκB cells were cultured in MEM with 200 ng/ml G418. For induction of differentiation, cells were cultured 3 days in the α-MEM containing 100 ng/ml RANKL. HEK293 cells were cultured in DMEM supplemented with 10% fetal bovine serum (FBS). Cells were incubated at 37 °C in a humidified atmosphere of 5% CO2.

Transfection of siRNA and plasmids were performed using Lipofectamine2000 from Life Technologies according to the manufacturer’s instructions.

### Immunoprecipitation and Western blotting

RAW264.7 cells were lysed in lysis buffer (150 mM Tris-HCl, 500 mM NaCl, 1% TritonX-100, 1× proteinase inhibitor cocktail, 1 mM sodium fluoride and 1 mM sodium orthovanadate, pH 7.6) for 30 min on the ice. After centrifugation at 12000rpm for 30 min at 4 °C, 2 mg of whole cell lysate were precleared with non-immunizedIgG and protein A/G Sepharose (millipore) for 30 min, The precleared lysates were then incubated with specific antibodies at 4 °C overnight. Protein A/G sepharose beads were then added, and incubated for further 4 hours. The beads were collected by centrifugation and washed three times with washing buffer (150 mM Tris-HCl, 500 mM NaCl, 1% Triton-X, pH 7.6), and the immune complexes were denatured with sample buffer containing 1 × SDS loading buffer for 10 min at 95 °Cand analyzed by Western blotting.

Western blotting was performed as described elsewhere. Briefly, proteins were resolved by SDS-PAGE and then transferred onto 0.2 μm PVDF membranes. Membranes were blocked with 10% skim milk in Tris-buffered saline-Tween 20 (TBS-T) for 1 hour, and then probed with primary antibodies diluted in 5% milk in TBS-T overnight at 4 °C. Membranes were washed three times with TBS-T and then incubated with secondary antibodies at RT for 1 hour. Specific protein bands were then visualized by chemiluminescence using Supersensitive ECL Chemiluminescent Kit. Phos-tag gel PAGE was performed according to the manufacturer’s instruction. The quantification of the protein bands was performed using *ImageJ* software.

### RNA interferences assays

For siRNA experiments, RAW264.7 cells were transfected in suspension with 100 nM GSK3β siRNA, hnRNPK siRNA or no target siRNA. Briefly, 2 × 10^5^ cells in a 200 μl suspension were mixed with 200 ul opti-MEM plus 5 μl lip2000 and incubated at RT for 10 min, then seeded in a six-wells plate with 200 μl complete medium. For an effective effect of hnRNPK knockdown, a mixture of two siRNA with a final concentration of 100 nM was used.

The sense and antisense sequences of siRNA used in this study are as follows: GSK3β-siRNA: 5′-AAGUAAUCCACCUCUGGCUACTT-3′, 5′-GUAGCCAGAGG UGGAUUACUUTT-3′; hnRNPK-siRNA-1: 5′- UAUUAAGGCUCUCCGUACATT -3′,5′-UGUACGGAGAGCCUUAAUATT-3′; hnRNPK-siRNA-2:5′-CCUUAUGAUCCCAACUUUUTT-3′, 5′-AAAAGUUGGGAUCAUAAGGTT-3′).

Lentivirus-packing shRNAs were used for effective knockdown of GSK3β and hnRNPK in BMM cells. lentiviruses bearing GSK3β, hnRNPK and non-target control shRNAs were provided by GeneChem (China), and used to infect BMM cells for 48 hours according to manufacturer’s instruction. The sequences of shRNA are as following:

GSK3β: CCGGCCACAGAACCTCTTGTTGGATCTCGAGATCCAACAAGAGGTTCTGTGGTTTTTG,

hnRNPK: CCGGCAGTGCTGATATTGAGACGATCTCGAGATCGTCTCAATATCAGCACTGTTTTTG.

Non-target control: CCGGTTCTCCGAACGTGTCACGTACGTGACACGTTCGGAGAATTTTTG.

### Reverse Transcription-PCR

Total RNA was extracted from cultured cells using Trizol, and the DNA was removed by the recombinant DNaseI. cDNA was prepared from 1 μg of total RNA, using reverse transcriptase with iScript^TM^ cDNA synthesis kit in accordance with the manufacturer’s protocol. All PCR was carried out by using SsoFast TMEvaGreen Supermix, using cycling parameters 95 °C, 15 seconds; 60 °C,15 seconds; and 72 °C,20 seconds for 39 cycles, with the following primers:

GSK3β (forward: 5′-GGCAGCATGAAAGTTAGCAGA-3′, reverse: 5′-GGCGACCAGTTCTCCTGAATC-3′);

hnRNPK (forward: 5′-CAATGGTGAATTTGGTAAACGCC-3′,

reverse: 5′-GTAGTCTGTACGGAGAGCCTTA-3′);

NFATc1 (forward: 5′-TGCTCCTCCTCCTGCTGCTC-3′,

reverse: 5′-CGTCTTCCACCTCCACGTCG-3′);

DC-STAMP (forward: 5′-GGAAGTTCACTTGAAACTACGTGGA-3′,

reverse: 5′-AGACACACTGAGACGTGGTTTAGGA-3′);

Cathepsin K (forward: 5′-GGCAGCATGAAAGTTAGCAGA-3′,

reverse: 5′-GCCTCCAGGTTATGGGCAGA-3′);

TRAP (forward: 5′-CAGCAGCCAAGGAGGACT AC-3′,

reverse: 5′-ACATAGCCCACACCGTTCTC-3′);

MMP9 (forward: 5′-GTTTTTGATGCTATTGCTGAGATCCA-3′,

reverse: 5′-CCCACATTTGACGTCCAGAGAAGAA-3′);

GAPDH: (forward: 5′-ACTTTGTCAAGCTCATTT CC-3′,

reverse: 5′-TGCAGCGAACTTTATTGATG-3′); ACTB (forward: 5′-ACGTGGA CATCCGCAAAG-3′,

reverse: 5′-GACTCGTCATACTCCTGCTTG-3′).

### Luciferase Reporter Assay

RAW264.7-NFκB cells were transfected with hnRNPK siRNA. 24 hours after transfection, 100 ng/mL RANKL or 10 mM LiCl was added. After 12 hours, cells were harvested and lysed. Luciferase activity was then measured by multifunctional micro firefly luciferase assays using Luciferase Reporter Gene Assay kit according to manufacturer’s protocol.

### Confocal Immunofluorescence

RAW264.7 cells cultured on the glass coverslips were washed with phosphate buffer saline, fixed with 4% paraformaldehyde in phosphate-buffered saline for 30 min at Room temperature, washed with 2 mg/ml glycine in phosphate-buffered saline, permeabilized with 0.2% TritonX-100 for 10 min, and blocked with 10% goat serum in phosphate-buffered saline for 60 min. The cells were then incubated with primary antibodies diluted in 2% goat serum phosphate-buffered saline overnight at 4 °C.After extensive washes with washing buffer (0.05% Tween-20, 1%BSA, phosphate-buffered saline), cells were incubated with fluorescent secondary antibodies for additional 60 min in the dark at room temperature, then washed again with washing buffer. The nuclei were counterstained with 4′, 6-diamidino-2-phenylindole (Sigma) 10 min and wash with phosphate-buffered saline. Images were acquired using confocal microscopy.

### TRAP staining

RAW264.7 or BMM cells cultured in 96-well plate were washed with phosphate buffered saline (PBS), fixed in 4% Paraformaldehyde for 20 minutes at room temperature, and washed thrice with PBS. 100 μl TRAP staining solution was added for incubation at 37 °C until cells were correctly stained (approximately 20 minutes after). TRAP staining solution was removed, and cells were washed trice with PBS, Stored plates at 4 °C with PBS in wells. Samples were examined under an Olympus Fluoview 500 microscope.

### Bone Marrow Derived Cells

The male mice with 6 weeks were sacrificed by cervical dislocation. Femur was extracted and muscle tissue was cleared. Intact bones were disinfected in 75% ethanol for 1 minute, washed twice with DPBS. Both ends of the femur were cut with scissors, and bone marrow was flushed out with Ca^2 + ^ and Mg^2 + ^ free DPBS by using a 25-gauge needle. After repeated pipetting to break cell aggregates, cells were filtered with 70 μm nylon mesh to remove debris, then spin down and gently resuspended with 2 ml ACK (Ammonium-Chloride-Potassium) Lysing Buffer for 2 min before adding 10 ml DMEM. Cells were washed again with DMEM and resuspended in culture medium and counted. Cells were grown in macrophage differentiation medium M-CSF (10 ng/ml) for 3 days; then fed with M-CSF (10 ng/ml) and RANKL (100 ng/ml DMEM) for 3 other days. All experiments were carried out in accordance with the approved guidelines and all animal experimental protocols were approved by the animal experimental ethics committee of Jinan University.

### *In vitro* bone resorption assay

The BMM cells were seeded at 10000 cells/slice on the surface of bone slices placed in the wells of 96-well plate, and incubated at 37 °C and 5% CO2 for 3 days with DMEM containing 10 ng/ml M-CSF. Lentiviral shRNA against either GSK3β or hnRNPK were added and incubated for 2 days before changing the medium with fresh α-MEM containing 10 ng/ml M-CSF and 100 ng/ml RANKL, and incubating for three other days. The bone slices were then washed with phosphate buffer saline, fixed in 2.5% Glutaraldehyde for 2 hours at room temperature. After 3 washes with phosphate-buffered saline, the dehydration was performed by subsequent incubations with ethanol with increasing concentration, ranging from 50% to 100% (with stepwise concentration of 10%), washed 3 times with isopentyl acetate for 10 min. Finally, after drying at critical point and spraying gold, absorption pits were observed by scanning electron microscopy. The total number and area of absorption pits on each bone slice was scored with the help of Adobe Photoshop CS5.

### Statistical analysis

Statistical analysis was carried using Student’s t test. Data were expressed as mean ± standard error of the mean of three independent experiments, unless otherwise stated, with P-value less than 0.05 considered significant.

## Additional Information

**How to cite this article**: Fan, X. *et al.* Cytoplasmic hnRNPK interacts with GSK3β and is essential for the osteoclast differentiation. *Sci. Rep.* **5**, 17732; doi: 10.1038/srep17732 (2015).

## Supplementary Material

Supplementary Information
