# Supplementary material for: Cytoplasmic hnRNPK interacts with GSK3β and is essential for the osteoclast differentiation
Source: Sci Rep. 2015 Dec 7;5:17732. doi: 10.1038/srep17732 (PMC4671015; doi:10.1038/srep17732)

Cytoplasmic hnRNPK interacts with GSK3β and is essential for the osteoclast differentiation

Xiaoqin Fan[1#], Haiting Xiong[1#], Jinmei Wei[1], Xuejuan Gao[1], Yuan Feng[1], Xiaohui Liu[1], Gong Zhang[1], Qing-Yu He[1,]*, Jiake Xu[2,]*, Langxia Liu[1,]*

Supplementary figure

Figure S1

Uncropped blots for the images shown in Fig. 1, 2, 3, 4, and 7.

All SDS-PAGE were run under the same experimental conditions with adequate durations, except for the Phos-tag PAGE that were performed according to the instructions of Wako Pure Chemical Industries, Ltd (Japan). In order to economize the reagents and simultaneously reveal several proteins migrated in a same gel, after membrane transfer, the membranes were generally cut into slices bearing the protein bands of interest whose position in the membrane were approximately determined according to their molecular weight, and incubated separately with the appropriate antibodies for the revelation. (a) Uncropped blots for the images shown in Fig. 1a. (b) Uncropped blots for the images shown in Fig. 1b. (c) Uncropped blots for the images shown in Fig. 1c. (d) Uncropped blots for the images shown in Fig. 2c (e) Uncropped blots for the images shown in Fig. 2d. (f) Uncropped blots for the images shown in Fig. 3a. (g) Uncropped blots for the images shown in Fig. 3c. (h) Uncropped blots for the images shown in Fig. 4d. (i) Uncropped blots for the images shown in Fig. 7a.

Fig.S1a

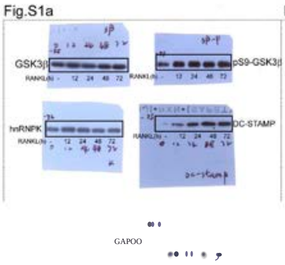

Fig.S1b

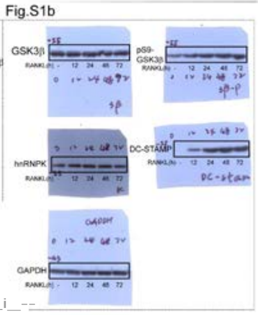

Fig.S1c

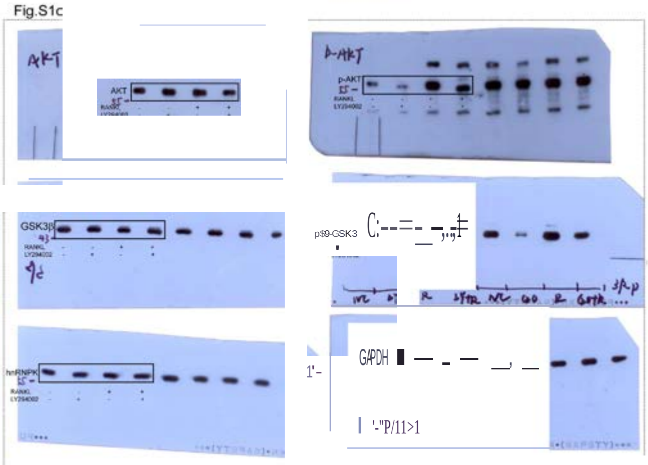

Fig.S1d

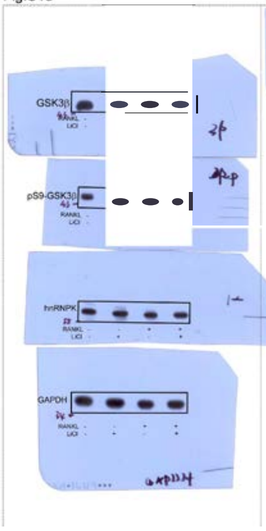

Fig.S1e

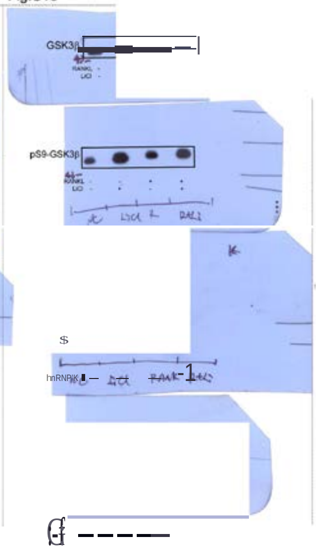

Fig.S1f

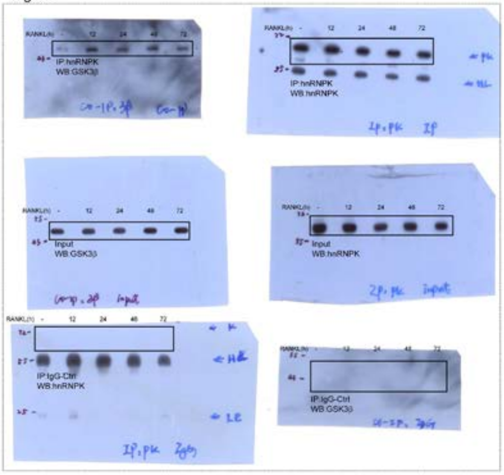

Fig.S1g

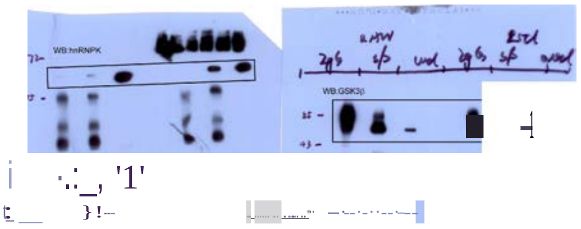

Fig.S1h

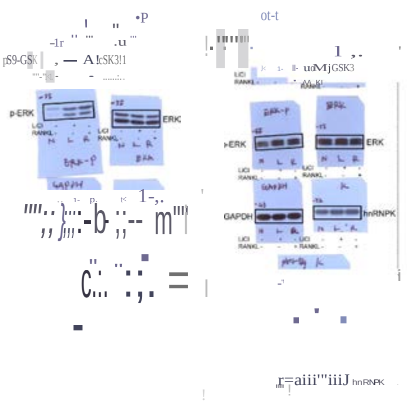

Fig.S1i

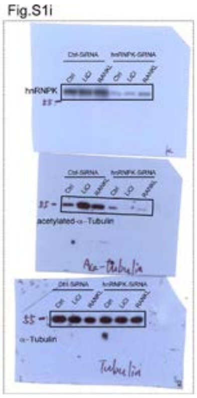

Supplement: Supplementary Information [file srep17732-s1.pdf]
